# Cerebellar Purkinje Cells Generate Highly Correlated Spontaneous Slow-Rate Fluctuations

**DOI:** 10.3389/fncir.2017.00067

**Published:** 2017-09-20

**Authors:** Ying Cao, Yu Liu, Dieter Jaeger, Detlef H. Heck

**Affiliations:** ^1^Department of Anatomy and Neurobiology, University of Tennessee Health Science Center Memphis, TN, United States; ^2^Department of Biology, Emory University Atlanta, GA, United States

**Keywords:** rate coding, rate correlation, cerebellar cortico-nuclear interaction, Purkinje cell activity, awake mouse, cerebellar nuclei, paired single unit recordings

## Abstract

Cerebellar Purkinje cells (PC) fire action potentials at high, sustained rates. Changes in spike rate that last a few tens of milliseconds encode sensory and behavioral events. Here we investigated spontaneous fluctuations of PC simple spike rate at a slow time scale of the order of 1 s. Simultaneous recordings from pairs of PCs that were aligned either along the sagittal or transversal axis of the cerebellar cortex revealed that simple spike rate fluctuations at the 1 s time scale were highly correlated. Each pair of PCs had either a predominantly positive or negative slow-rate correlation, with negative correlations observed only in PC pairs aligned along the transversal axis. Slow-rate correlations were independent of faster rate changes that were correlated with fluid licking behavior. Simultaneous recordings from PCs and cerebellar nuclear (CN) neurons showed that slow-rate fluctuations in PC and CN activity were also highly correlated, but their correlations continually alternated between periods of positive and negative correlation. The functional significance of this new aspect of cerebellar spike activity remains to be determined. Correlated slow-rate fluctuations seem too slow to be involved in the real-time control of ongoing behavior. However, slow-rate fluctuations of PCs converging on the same CN neuron are likely to modulate the excitability of the CN neuron, thus introduce a possible slow modulation of cerebellar output activity.

## Introduction

Purkinje cells (PCs), the principal neurons of the cerebellar cortex, generate action potentials at baseline rates between 30 and 200 spikes/s (Thach, 1970; Bryant et al., 2010; Cao et al., 2012a). PCs form GABAergic synapses on neurons in the cerebellar nuclei (CN), which constitute the cerebellar output neurons. CN neurons themselves are spontaneously active, albeit at lower rates than the PC (Lu et al., 2013). CN neurons receive converging inputs from about 50 PC (Person and Raman, 2012). How the activity of CN neurons is controlled by the converging inputs from PC is a question of central importance to understanding the neuronal principles of cerebellar function. *In vitro* and modeling studies have focused mostly on temporal synchronization of PC simple spike activity within millisecond time windows (Gauck and Jaeger, 2000; Steuber et al., 2011; Person and Raman, 2012). PC activity can indeed be highly synchronized at millisecond precision during specific phases of a behavior (Heck et al., 2007). Traditionally, the analysis of cerebellar spike trains and their relationship with sensory and motor events is based on millisecond temporal resolution of spike times or of the length of inter-spike intervals (instantaneous rate). PCs and CN neurons continually fire at high rates, and this ongoing spike activity shows slow, spontaneous rate fluctuations at a time scale of ~1 s, that are not obviously linked to any sensory or motor event. Here we asked whether these slow-rate fluctuations are independently generated in each individual PC or, whether neighboring PCs show similar slow-rate changes, which would suggest that slow-rate fluctuations are controlled by common inputs to multiple cells. We analyzed single unit simple spike trains from pairs of PCs recorded simultaneously in the anterior cerebellar vermis or from simultaneous recordings of a single unit vermal PC and a single unit fastigial nucleus (FN) neuron. By aligning the pair of recording electrodes along either the transversal or sagittal axis of the cerebellar cortex we also distinguished between pairs of PCs aligned with the direction of parallel fibers (transversal pairs) or with the direction of inhibitory axons of molecular layer interneurons (sagittal pairs). Additional functional relevance for the differentiation between sagittal and transversal alignment in the cerebellar cortex comes from findings showing that the communication in the olivo-cerebellar system is organized in sagittal zones (Scheibel, 1977; Ruigrok, 2011) and that molecular markers, such as zebrin II (Sillitoe and Hawkes, 2013; White et al., 2014), divide the cerebellum into parallel sagittal zones within which PCs have been shown to have different physiological properties (Ebner et al., 2012). In the terminology of Eccles et al. (1967) the transversal pairs are “on beam” neighbors as they receive excitatory inputs from overlapping populations of parallel fibers, whereas sagittal PC pairs are “off beam” neighbors who do not receive shared excitatory inputs.

## Materials and Methods

### Animals

Experiments were performed on male adult C57BL/6J (B6) mice (18–25 g, Jackson Laboratories, Bar Harbor, ME, USA). All mice used in this study were raised in the AAALAC accredited animal facilities at the University of Tennessee Health Science Center. This study was carried out in accordance with the recommendations of University’s Animal Care and Use Committee. The protocol describing all experimental procedures involving mice were approved by the University’s Animal Care and Use Committee. Principles of laboratory animal care (NIH publication No. 86-23, rev. 1996) were followed.

### Surgery

A detailed description of the surgical and experimental procedures has been given previously (Bryant et al., 2009, 2010). In short: mice were initially anesthetized with 3% isoflurane in oxygen (Ohio isoflurane vaporizer, Highland Medical Equipment, Deerfield, IL, USA) in an incubation chamber and then transferred to a stereotaxic device. Anesthesia was maintained through a nose cone with 1%–2% isoflurane in oxygen during surgery. The depth of anesthesia was adjusted so that the mice failed to show a reflex withdrawal of the hind paw to a strong pinch. Rectal temperature was maintained at 37°C–38°C with a servo-controlled heat blanket (FHC, Bowdoinham, ME, USA). Standard surgical techniques were used to secure three small machine screws in the skull (1/8’ dome head, 0.8 mm diameter, 2 mm long; Small parts, Miami Lakes, FL, USA). A craniotomy (4–5 mm diameter) was performed to expose vermis and simple lobule of the right cerebellar hemisphere. The exposed dura was covered with triple antibiotic ointment (Walgreens, Deerfield, IL, USA) to keep it moist and to reduce infection risk. A cylindrical plastic chamber was placed over the craniotomy. A custom-made head post was placed on the skull and the chamber and head post were secured to the skull screws with dental acrylic (Teets methyl methacrylate denture material, CoOral-Lite Mfg. Diamond Spring, CA, USA). The plastic chamber was then completely filled with Triple Antibiotic Ointment. While still under anesthesia mice were injected subcutaneously with 2.5 mg/kg Torbugestic (Fort Dodge, IA, USA) in 0.5 ml ringer solution to alleviate pain and supply fluid following and 24 h after the surgery. A post-surgical recovery period of 3–4 days was observed before electrophysiological experiments began.

### Electrophysiology and Behavior

**Figure 1:**
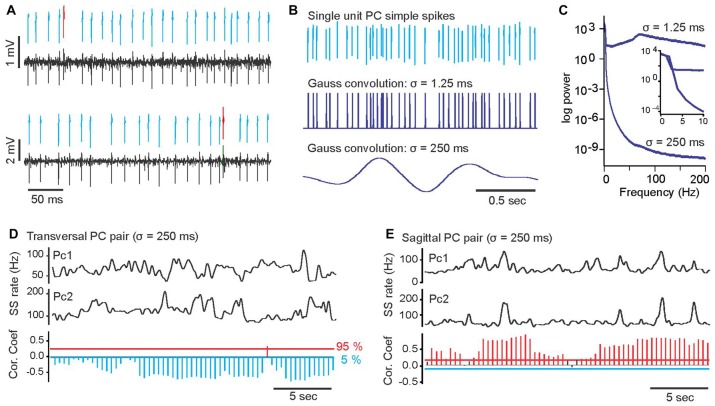
Examples of a paired, single unit Purkinje cells (PC) recording, transformation of PC simple spike trains into continuous rate functions and slow-rate correlations of PC pairs. **(A)** Example of simultaneously recorded spike trains of two single unit PCs. Black bottom traces are band-pass filtered raw voltage recordings. Above the raw voltage trace, printed in blue and red are extracted waveforms of simple and complex spikes, respectively. **(B)** Transformation of PC simple spike trains into continuous rate functions by convolution of simple spikes (top trace) with a Gaussian kernel with *σ* = 1.25 ms (middle trace) and a with Gaussian kernel with *σ* = 250 ms (bottom trace). **(C)** Power spectra of the PC spike train in **(B)** after convolution with *σ* = 1.25 ms (upper trace) and *σ* = 250 ms (lower trace). Insert shows enlarged section of power spectrum for frequencies from 0 Hz to 10 Hz. **(D)** Top two traces show smoothed (*σ* = 250 ms) simple spike trains of two simultaneously recorded PCs (PC1, PC2) with predominantly negative slow-rate correlations. The bottom histogram shows the results of time-resolved correlation analysis of the smoothed rate traces above. Each bar in the histogram represents by its length the magnitude of the peak correlation within a 5 s sliding window. The direction and color of the bars indicate the sign of the correlation. Blue, downward bars represent significant negative correlations and red upward bars represented significant positive correlations. Black bars represent correlations that did not reach statistical significance. Horizontal red and blue lines mark 95 and 5 percentile boundaries of the surrogate distribution, respectively. **(E)** Same plot format as in **(D)**, showing an example of a PC pair with predominantly positive slow-rate correlation.

During electrophysiological experiments, the mice’s heads were fixed to a metal holder via the head post and the body was loosely covered with a plastic tube to limit body movements (Bryant et al., 2009; Cao et al., 2012a,b). The recording chamber was cleaned and filled with Ringer’s solution. Two recording electrodes (glass-insulated tungsten/platinum, 80 μm; O.D, impedance 3–7 MΩ), spaced 305 μm apart were inserted into the cerebellum using a computer-controlled microdrive (System Eckhorn, Thomas Recording, Germany). The electrode pair’s orientation was either aligned with the transversal or the sagittal axes, corresponding to an “on-beam” or “off-beam” recording configuration, respectively, in the terminology introduced by Eccles et al. (1967). Electrodes were advanced until two stable single unit PCs were isolated (Figure 1A). PCs were identified based on firing characteristics such as the presence of complex spikes as well as sustained high frequency simple spike firing (Figure 1A; Thach, 1970; Bryant et al., 2010; Cao et al., 2012a). The number of paired recordings analyzed was mainly limited by the technical difficulty of maintaining stable paired single unit recordings, particularly during licking behavior. We only used data with at least 1 min of stable recordings for both units. Typical durations of stable recordings were between 1 min and 3 min.

The FN is the most medial of the cerebellar nuclei. It receives input from PCs in the vermis. All PC recordings analyzed here where performed in the vermis. In order to obtain simultaneous PC and FN neuron recordings, one of seven electrodes was lowered stereotaxically into the FN, which is located between −6 mm to −6.5 mm posterior to the Bregma, 0.4–1 mm lateral to the midline and 2–2.6 mm inferior to Bregma (Paxinos and Franklin, 2001). Nuclear activity was identified by electrode depth, the electrode passing through an area without spiking activity (i.e., the white matter embedding the FN) before reaching the nucleus, and finally by the presence of sustained spiking (8–50 Hz) without the occurrence of complex spikes. Raw voltage signals were band-pass filtered (200–8 kHz) and amplified (total: 230×) using a filter-amplifier (FA32, Multichannel Systems, Germany). Filtered and amplified voltage signals were digitized (16 bit A/D converter, sampling rate >20 kHz) and stored on a computer hard disk using a CED power1401 and Spike2 software (both Cambridge Electronic Design, UK).

After each experiment the cranial recording chamber was rinsed with sterile ringer solution and filled with triple antibiotic ointment, before animals were returned to the home cage. During the last two experimental sessions, 2–3 small electrolytic lesions (5 μA/10 s) were formed in stereotaxically defined locations using the head post as reference.

To prepare the brains for anatomical verification of recording sites mice were deeply anesthetized with intraperitoneal injection of Avertin (Tribromoethanol, 500 mg/kg) and transcardially perfused with 0.01 M phosphate-buffered saline (PBS), followed by 4% formaldehyde. After the perfusion the cerebellum was removed and fixed in 4% formaldehyde for 24 h. The cerebellum was cut into 50 μm coronal sections, which were mounted and stained with cresyl violet. The location of lesion sites was determined using a stereotaxic atlas of the mouse brain (Paxinos and Franklin, 2001).

### Data Analysis

Single unit PC were identified based on spike rate (30–200 Hz), the presence of simple and complex spikes and a minimal 3 ms refractory period in simple spike activity. Simple and complex spikes were separated based on differences in spike waveform shapes using off line spike sorting software (Spike2, Cambridge Electronic Design, UK). All analysis of PC activity was performed on simple spikes. Single unit FN neurons were identified based on the criteria described above. Recordings of single unit PC or FN neurons that were stable for 60 s or longer were chosen for further data analysis. We analyzed 12 pairs of PC aligned along the transversal axis (on-beam), 11 PC pairs aligned along the sagittal axis (off-beam) and 11 PC-FN pairs. We did not attempt to find connected PC-FN pairs, as the probability of success would have been extremely low. In none of the PC-FN pairs we recorded did the spike-time cross-correlations indicate a postsynaptic inhibition of the CN neuron by the simultaneously recorded PC. We thus interpreted our data as representing spike activities in PC-FN pairs that were not synaptically connected. However, the PCs were located in a cerebellar cortical area that projects to the nucleus from where the CN neuron was recorded.

Spikes trains were convolved with Gaussian kernels with standard deviation (σ) values of either *σ* = 250 ms to yield a smoothed slow-rate function, or with *σ* = 1.25 ms to preserve the spike temporal information (Figure 1B). Power spectra of smoothed rate functions were calculated using FFT based script functions in Spike 2 (Cambridge Electronic Design, UK; Figure 1C).

We used cross-correlation analysis to calculate the correlation coefficient as a quantitative measure for determining the strength and sign of slow-rate and spike-time correlations for spike trains convolved with *σ* = 250 ms and *σ* = 1.25 ms Gaussian kernels, respectively. While the correlation coefficient provides a quantitative measure of temporal correlation of two functions, it does not, by itself allow conclusions about whether the measured correlation is significantly above or below a chance, if compared to similar functions. Thus, in order to determine statistical significance of correlation results we applied bootstrap statistics (Diaconis and Efron, 1983; Efron and Tibshirani, 1993). To this end we generated surrogate correlation histograms by repeatedly shifting one of the two spike sequences by random time intervals and re-calculating the correlation. This process, also known as “shuffling data”, was repeated 100 times, resulting in 100 surrogate correlation histograms, which were bin-wise sorted by rank. Within each bin the median, 95th and 5th percentile of the distribution were determined. Raw correlation values crossing the 95th or 5th percentile threshold were considered significant at *p* < 0.05 and interpreted as meaning that the correlation of the two spike trains was higher than could be expected by chance.

To be able to compare correlation values between mice, we quantified cross-correlation peak values between single-unit spike trains by expressing correlation peak values with a ratio equivalent to *z*-scores calculated for normal distributions. To this end we first determined the difference between each raw correlation’s peak value (irrespective of whether the correlation was positive or negative) and the corresponding median of the surrogate data. We then calculated the difference between that surrogate data median and the corresponding 95th percentile value. Finally, we divided the difference between the peak correlation value and the surrogate median by the difference between the surrogate median and 95th percentile values. This ratio was termed *R*max (This procedure is similar to the calculation of *Z*-scores for normal distributions). *R*max values obtained with *σ* = 250 ms and *σ* = 1.25 ms Gaussian kernels were compared using Student’s *t*-test.

To investigate whether and how spike activity correlations between PC pairs and PC-FN pairs varied over time, we calculated time-resolved cross-correlations (Figures 1D,E). Correlations were calculated within 5.0 s time windows and the peak correlation values within each window were assigned to the time corresponding to the center of the window. Windows were then shifted in 0.5 s increments and the calculation repeated for every shift. Bootstrap statistical evaluation as described above (Diaconis and Efron, 1983; Efron and Tibshirani, 1993) was again used to determine whether spike rate correlations within any given time window significantly deviated from expectation based on the assumption that the two spike trains are independent. If correlation values within a 5 s window crossed the 95 and 5 percentile threshold of the surrogate distributions, correlations were considered significant at *p* < 0.05 and the result was assigned to the experimental time corresponding to the center of the sliding window.

To represent the results graphically, we plotted discrete bars along the time axis of the recording with the length of each bar corresponding to the correlation value within the corresponding time window and with the color and direction of each bar indicating the sign of the correlation and whether it was significant. Positive correlations within a window are indicated by blue, upward projecting bars, negative correlations by red, downward projecting bars. Black bars mark correlations that were not significant.

In order to determine whether slow-rate correlations were independent of the performance of a behavior represented by a pair of PCs millisecond-scale spike activity changes, we compared slow-rate correlations during rest and during performance of fluid licking behavior in five pairs of PCs, where each individual PC showed a significant correlation between simple spike firing and licking behavior at the *σ* = 1.25 ms time scale. We and others have previously shown that licking behavior is widely represented in the medial cerebellar cortex (Welsh et al., 1995; Bryant et al., 2010). Licking in mice occurs at a rate of about 10 Hz (Boughter et al., 2007). In order to determine whether a PC had lick-related simple spike activity we cross-correlated spike times with lick-times and used bootstrap procedures to evaluate whether spike-licking correlations were significant (Bryant et al., 2010; Cao et al., 2012a). Next, we calculated time-resolved slow-rate correlations at the *σ* = 250 ms time scale during rest and during licking behavior. Slow-rate correlation values measured during an uninterrupted train of licks with no inter-lick-interval longer than 300 ms were averaged and assigned to “licking behavior”. Correlation values measured during periods when the mouse was not licking were assigned to the “resting” state. The average slow-rate correlation values during licking and resting states measured in 5 mice were not normally distributed and were compared using Mann-Whitney *U* test.

## Results

PCs and CN neurons of the cerebellum generate action potentials at high, sustained rates independent of the behavioral state of the animal (Thach, 1970; Bryant et al., 2010; Cao et al., 2012a). To determine whether the simple spike activity of pairs of PCs and of individual PCs and CN neurons are correlated, we performed simultaneous recordings of single unit spike activity in pairs of PCs (Figure 1A) and PC-FN neuron pairs and performed cross-correlation analyses of simultaneously recorded spike trains at two different temporal resolutions. In addition to analyzing spike activity at the standard millisecond temporal resolution of spike duration, we also performed an analysis of spike rate fluctuations at a more than 100 times slower time scale. High and low time resolution versions of each spike train were created by convolving spikes with a Gaussian kernel with a standard deviation (σ) of either 1.25 or 250 ms. With a σ of 1.25 ms the temporal information of individual spike times is preserved whereas a σ of 250 ms creates a smooth rate function that does not retain details of individual spike times (Figure 1B). Comparison of the power spectra of the two rate functions illustrates the strong decline in power for frequencies above ~4 Hz (Figure 1C). In the following we will refer to the correlations at these two time scales as spike-time and slow-rate correlations.

The network architecture of the cerebellar cortex has two functionally distinct and perpendicular axes, defined by the projections of excitatory and inhibitory axons (Braitenberg and Atwood, 1958; Palay and Chan-Palay, 1974). Along the transversal axis run the parallel fibers, which provide excitatory synaptic input to PC and molecular layer interneurons, including stellate and basket cells. Axons of molecular layer interneurons, which provide inhibitory input to PCs, project along the sagittal axis, i.e., perpendicular to the direction of parallel fibers. Because of this well-ordered arrangement, PCs aligned along the transversal axis receive excitatory inputs from overlapping groups of parallel fibers, whereas PCs aligned along the sagittal axis receive excitatory inputs from different sets of parallel fibers but may share inhibitory inputs (Eccles et al., 1967).

Convolution of PC spike trains with Gaussian kernels of *σ* = 250 ms revealed that slow simple spike rate changes of pairs of PCs were highly correlated. These slow-rate correlations were of such magnitude and stability that they could be determined directly from visual inspection of the smoothed spike rate traces (Figures 1D,E). Smoothed spike traces of a pair with negative slow-rate correlations appeared as mirror images of each other (Figure 1D), whereas the traces of positively correlated spike trains had were very similar time courses (Figure 1E). Time-resolved correlation analysis using sliding windows (see “Materials and Methods” Section) revealed that correlation-strength varied over time, but the sign of the correlation remained either predominantly positive or negative depending on the specific pair of PCs (Figures 1D,E, bottom histograms). Thus, the average slow-rate correlations for each pair of PCs, calculated over the duration of the observation (1–3 min) was either significantly positive or negative, with only one pair out of 23 showing no significant slow-rate correlation.

**Figure 2:**
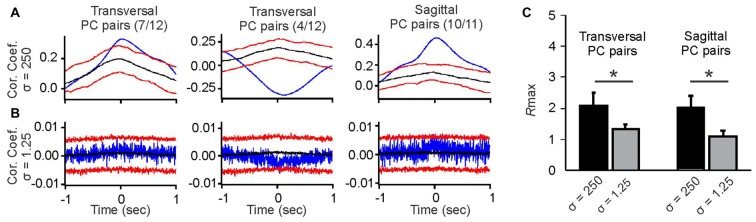
Comparison of averaged slow-rate and spike-time correlations of simple spike activity of pairs of PCs aligned along the transversal or sagittal axis. **(A)** Example cross-correlograms of slow spike rate activity in pairs of PCs smoothed with a Gaussian kernel with *σ* = 250 ms. Blue traces are raw cross-correlations, black traces represent the medians of the surrogate distributions, and red traces represent the 95 and 5 percentile boundaries of surrogate distributions. Raw correlations exceeding either boundary were considered significant at *p* ≤ 0.05. The average slow-rate correlations of PC pairs aligned along the transversal axis were either positive (left histogram) or negative (middle histogram), with the exception of one pair, whose slow-rate fluctuations were not correlated (data not shown). The average slow-rate correlations among PC pairs aligned along the sagittal axis were all found to be positive (right histogram), with the exception of one pair, whose slow-rate fluctuations were not correlated (data not shown). **(B)** Example cross-correlograms of simple spike activity of pairs of PCs smoothed with a Gaussian kernel with *σ* = 1.25 ms. Blue lines represent raw spike-time correlations. Colors of lines are the same as in **(A)**. Spike-time correlations in the examples shown do not reach significance. **(C)** Comparison of *R*max values as a measure of correlation strength for slow-rate vs. spike-time correlations in sagittal and transversal PC pairs. Asterisks indicate significant differences between *R*max value distribution means at *p* < 0.05 (Student’s *t*-test).

We found PC pairs with positive slow-rate correlations along both spatial orientations (transversal and sagittal), while negative slow-rate correlations were only observed in PC pairs aligned along the transversal axis (Figure 2A, upper row). Out of 12 transversal PC pairs seven showed positive and four showed negative slow-rate correlations. One pair showed no significant slow-rate correlation. Out of 11 PC pairs along sagittal axis 10 showed positive slow-rate correlations and one pair showed no significant slow-rate correlation (Figure 2A, upper row).

In contrast to the slow-rate correlations, spike-time correlations of the same PC pairs were weak or non-significant, irrespective of alignment along the sagittal or transversal axis. The example correlations shown in Figure 2A do not exceed the 95 and 5 percentile boundaries with *R*max values remaining close to 1 (Figure 2B). *R*max values for slow-rate correlations were significantly higher than those for spike-time correlations for both transversally and sagittally aligned pairs (Figures 2B,C).

**Figure 3:**
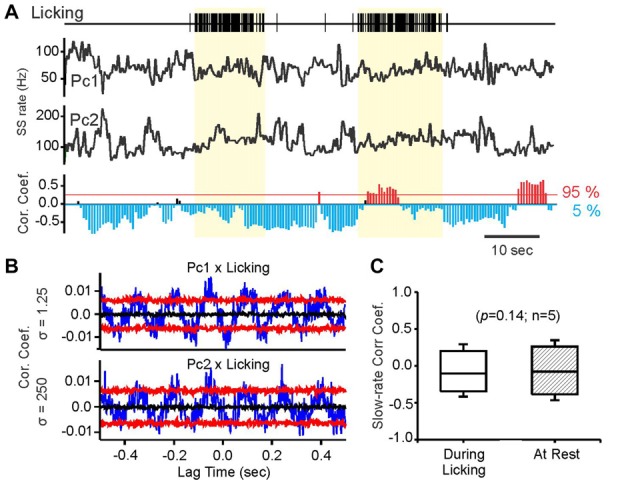
Slow-rate correlations persist independent of behavior related simple spike rate modulations. **(A)** Vertical tick marks in top trace represent individual licks recorded during fluid licking behavior. Black traces labeled PC1, PC2 represent smoothed simple spike activity (Gauss convolution, *σ* = 250 ms) of a pair of transversal PCs, recorded while the mouse performed fluid licking behavior. The yellow-shaded areas mark periods of uninterrupted licking. The bottom histogram shows the results of time-resolved cross-correlation analysis of the rate traces above. Each bar in the histogram represents by its length the magnitude of the peak slow-rate correlation within a 5 s sliding window. The direction and color the bars indicate the sign of the correlation, with significant negative correlations represented by blue, downward pointing bars and significant positive correlations by red, upward pointing bars. Non-significant correlations are represented by black bars. Bars are placed along the time axis at the center of the sliding window. **(B)** Cross-correlations of simple spike times with lick times for PC1 and PC2 showing significant correlations in both cells. Blue traces are raw cross-correlations, black traces represent the medians of the surrogate distributions, and red traces represent the 95 and 5 percentile boundaries of surrogate distributions. Raw correlations exceeding either boundary were considered significant at *p* ≤ 0.05. **(C)** Comparison of *R*max values for slow-rate correlations obtained during periods of uninterrupted licking (yellow shaded areas in **(A)** compared to equally long rest periods, without licking behavior.

Slow-rate correlations were not altered when the mouse performed fluid licking behavior represented in the simple spike activity of both PCs in the pair. Licking is a behavior widely represented in the cerebellar cortex (Welsh et al., 1995; Bryant et al., 2010). We recorded pairs of transversally aligned PCs with negative average slow-rate correlations while the mice licked water. Example slow-rate traces are shown in Figure 3A. PC pairs were only included in this analysis if both cells showed significant simple spike correlations with fluid licking at the millisecond time scale. A total of five PC pairs satisfied this criterion. Significance of the correlation between simple spike activity and licking was determined using bootstrap statistics of spike-time correlations. Examples of significant lick-spike cross-correlations for two simultaneously recorded PCs are shown in Figure 3B. There was no difference between slow-rate correlations measured during licking behavior compared to rest (Figure 3C, Mann-Whitney *U* test).

**Figure 4:**
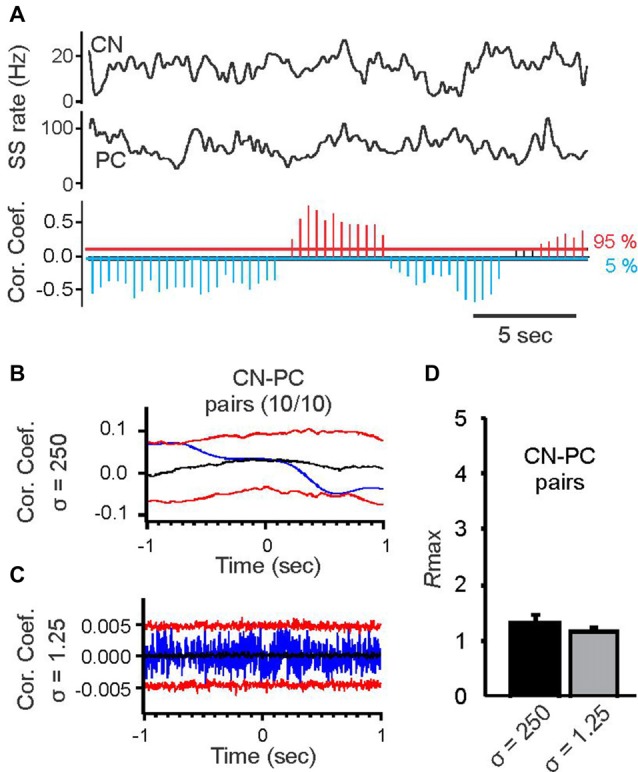
Cross correlation analysis of spike activity in simultaneously recorded PC pairs and pairs of PCs and cerebellar nuclear (CN) cells at two different time scales. **(A)** Top two black traces show spike activity of a CN neuron and a simultaneously recorded PC smoothed with a Gaussian kernel with *σ* = 250 ms. The histogram below shows time-resolved cross correlation results for the two smoothed rate functions (format of time-resolved correlation plot as in Figure 1C). Slow-rate correlation of PC and CN spike trains continually alternated between periods of positive and negative correlations. **(B)** Average slow-rate correlation of the PC-CN pair shown in **(A)**. **(C)** Average spike-time correlation of the PC-CN pair shown in **(A)**. Format of correlation plots in **(B,C)** same as in Figures 2A,B. **(D)** Average *R*max values for the slow-rate (*σ* = 250 ms) and spike time (*σ* = 1.25 ms) correlations of 10 PC-CN pairs. There was no significant difference between the *R*max values for the two time scales (Student’s *t*-test).

In order to determine if similarly strong correlations or anti-correlation existed between PCs and CN neurons we performed paired recordings from 10 pairs of PCs and FN neurons. These recordings were performed in awake, head-fixed mice at rest, i.e., no licking behavior was performed. In contrast to the PC pairs, where slow-rate correlations were predominantly positive or negative for each specific pair, slow-rate correlations between PC-FN pairs were continually changed between states of positive and negative slow-rate correlations, with each state lasting several seconds (Figure 4A). It is important to note that for the duration of each state correlations reached statistical significance, i.e., exceeded the 95 or 5 percentile boundaries (Figure 4A). But, when the average correlation was calculated over the 1–3 min time-course of the recording the resulting averaged slow-rate correlations were not significant. It is thus likely that the two alternating states of significant positive and negative correlations canceled each other out when correlations were averaged over a longer period of time. An example slow-rate correlation is shown in Figure 4B.

We also calculated spike time correlations for each PC—FN pair to determine whether they might be synaptically connected. In none of the 10 pairs did we find positive or negative spike time correlations. An example correlation is shown in (Figure 4C). Analysis of *R*max values for the average slow-rate and spike time correlations revealed no significant differences (Figure 4D).

## Discussion

Here we report that pairs of PCs in the awake mouse cerebellum express slow fluctuations in their simple spike rates which for any given pair of PCs are either highly correlated or anticorrelated. Slow-rate correlations between simultaneously recorded PCs and CN neurons were also highly correlated but the sign of that correlation continually altered between positive and negative. We did not attempt to obtain connected PC-CN pairs. Based on the low probability of obtaining such pairs by chance we assume that our PC-CN pairs were not connected.

When recording from PCs we oriented our electrodes such that we obtained pairs aligned either along the sagittal or the transversal axis. Slow-rate correlations of 10 out of 11 sagittal pairs of PCs were positive. One sagittal PC pair did not show a significant slow-rate correlation. Out of 12 transversal pairs seven showed a positive correlation and four showed a negative correlation of slow-rate simple spike activity. One sagittal PC pair showed no slow-rate correlation. The slow-rate correlations and anti-correlations of simple spike rates can be so pronounced, that they are directly visible in the raw spike rate traces, which is how we first discovered this novel aspect of cerebellar simple spike activity. An example of a highly anti-correlated slow-rate trace is shown in Figure 1D, and an example of a PC pair with strong positive slow-rate correlation is shown in Figure 1E.

We recorded from 23 pairs of PCs and found positive slow-rate correlations in 17/23 pairs (~73%), negative slow-rate correlation in four (~17%). Thus, 21/23 pairs or 91% showed significant slow-rate correlations of simple spike activity, which suggests that slow-rate correlations are very common, at least in the anterior vermis.

Possible clues as to what determines which slow-rate correlation pattern a PC will express come from the spatial relationship between pairs. Along the sagittal axis we only observed positive slow-rate correlation. By contrast, along the transversal axis seven pairs had positive and four pairs had negative slow-rate correlations. While our sample size is too small to draw a firm conclusion, our data suggest the possibility that the spatial relationship between PC pairs plays a role in determining the sign of their slow-rate correlation. Negative slow-rate correlations might be limited to or occur preferentially between pairs of PCs aligned along the transversal axis, i.e., along the direction of parallel fibers. This finding suggests that PCs might belong to distinct functional groups and that cells within the same sagittal zone belong to the same group.

This spatial distinction between sagittal and transversal directly relates to the subdivision of the cerebellum in parasagittal zones. Parasagittal zones are defined by the pattern of expression of more than a dozen of distinct molecular markers, which subdivide the cerebellar cortex into numerous rostrocaudally oriented bands, that have sharp boundaries can be several hundred micrometers wide (Hawkes et al., 1992; Cerminara et al., 2015). Multiple labs have demonstrated that PCs in different parasagittal zones have different physiological properties, including differences in excitability, synaptic plasticity and in the type of information they represent (Ebner et al., 2012; Graham and Wylie, 2012). Furthermore, the strength and spatial extent of inhibition by molecular layer interneurons has been shown to differ between parasagittal zones (Gao et al., 2006), which might alter the efficiency of feed forward inhibition along the beam of parallel fibers (Mittmann et al., 2005).

Thus, based on our current understanding on cerebellar sagittal zones, the assumption that PC pairs located in different parasagittal zones (i.e., transversal pairs) express negative slow-rate correlations, whereas PC pairs located in the same parasagittal zone (i.e., sagittal pairs) express positive slow-rate correlations would explain why we only found negative slow-rate correlations between transversal PC pairs.

It remains unclear where the observed slow-rate activity patterns originate and whether they are driven by sensory events, intrinsic processes, neuromodulatory inputs, or all of the above. Slow-rate modulations could be driven by mossy fiber inputs. White et al. (2014) have recently shown that zonal patterning and the pattern of mossy fiber innervation in the anterior cerebellum depend on PC activity, suggesting that zone specific slow-rate modulations of mossy fiber activity and the downstream PCs might interactively shape slow-rate correlations within and across parasagittal zones.

Simultaneous recordings from PCs and CN neurons showed that their slow-rate correlations continually switched from positive to negative correlations. We suggest two possible explanations for this observation. First, it is possible that slow-rate fluctuations in CN neurons are alternately dominated by inputs from two distinct sources. These two sources could be two distinct groups of PCs: one group whose slow-rates are correlated with the PC we recorded simultaneously with the CN neuron and another group of PCs whose slow-rate fluctuations are anticorrelated to the observed PC. If our above hypothesis is correct and PCs located in different parasagittal zones express anticorrelated slow-rate activity the transitions in CN-PC slow-rate correlations would reflect transitions of CN neuron functional connectivity between parasagittal zones.

A second possibility is that CN neuron slow-rate fluctuations are at some times driven predominantly by PCs (which would result in anti-correlated CN-PC slow-rates) and at other times by mossy fiber inputs that have the same slow-rate profile as the PCs, which, because of the excitatory drive mossy fibers have on CN neurons would result in a positive correlation between PC and CN slow-rates.

It remains to be determined where the slow-rate fluctuations originate in the first place. Electrophysiological studies in the visual system have shown that the temporal scale of spike correlations is related to the spatial extend of the observed network. Precise spike synchrony is limited to neurons within a 3 mm range but slower correlated fluctuations occur between neurons separated by as much as 10 mm (Smith and Kohn, 2008). Thus, slow correlations in the neocortex might reflect network activity at a larger spatial scale. This seems to fit with imaging studies which suggest that the neocortex is organized into large functional networks that express anticorrelated activity patterns in form of slow (<0.1 Hz), spontaneous fluctuations of blood oxygenation levels dependent (BOLD), both at rest and during behavior (Fox et al., 2005). It is thus possible that mossy fiber projections originating in two anticorrelated neocortical networks target two separate groups of PCs, driving highly correlated slow-rate fluctuations between PCs of the same group but highly anticorrelated fluctuations between the two PC groups. In this scenario, the anticorrelated slow-rate activity is generated outside of the cerebellum and independent of cerebellar cortical circuitry. If this were the case, it is still possible that slow-rate correlations are linked to cerebellar sagittal zones, if neocortical projections carrying correlated BOLD activity terminate in the same type of parasagittal zone and mossy fibers carrying anticorrelated BOLD activity terminating in different sagittal zones. Another possibility is that the responses of PCs located in different sagittal zones to correlated inputs are shaped differently based on the known zone-specific differences in the physiological properties of PCs (De Zeeuw et al., 2011; Ebner et al., 2012).

Our data suggest that slow-rate correlations are independent from faster rate changes that represent ongoing behaviors. We recorded from pairs of PCs that represented fluid licking behavior. The correlation coefficients for slow-rate correlations calculated during rest and fluid licking were the same, showing that the much faster rate fluctuations that represent licking can be generated independently of the correlated flow rate fluctuations. It is however, possible that behavior related output of CN neurons is modulated by PC slow-rate fluctuations, depending on whether the behavior occurs during a phase of positive or negative correlation between CN and PC slow-rate modulation.

In summary, our findings reveal a new aspect of cerebellar neuronal activity that occurs at a time scale too slow to be detected by data analyses commonly applied to identify behavior related neuronal signaling on a 1–100 ms time scale. While we do not yet understand the neuronal mechanisms underlying slow-rate correlations and don’t know their possible relevance for cerebellar function, they are observed in such a large proportion of PC pairs and the correlations and anticorrelations of such magnitude that investigation of PC and CN activity should take slow-rate changes into account.

## Author Contributions

DHH conceived the original idea; participated in part of the experiments; advised on data analysis and created custom scripts. DHH, YC, YL and DJ designed the experiments. YC and YL performed the experiments and performed the majority of data analysis. DHH, YC and DJ wrote the article. YL contributed to the writing.

## Conflict of Interest Statement

The authors declare that the research was conducted in the absence of any commercial or financial relationships that could be construed as a potential conflict of interest.
